# Work-related stress and quality of life in nurses during the Covid-19 pandemic

**DOI:** 10.15649/cuidarte.3042

**Published:** 2024-05-21

**Authors:** María Angélica Díaz Hernández, Angie Paola Gorrostola Camargo, Darío Mendoza Romero

**Affiliations:** 1 Universidad del Sinú, Montería, Colombia. mariadiazh@unisinu.edu.co Universidad del Sinú Universidad del Sinú Montería Colombia mariadiazh@unisinu.edu.co; 2 IPS Cuidado seguro en casa, Montería, Colombia. anggie 2812@hotmail.com IPS Cuidado seguro en casa Montería Colombia anggie 2812@hotmail.com; 3 Fundación universitaria del Areandina, Bogotá, Colombia. dmendoza16@areandina.edu.co Fundación Universitaria del Área Andina Fundación universitaria del Areandina Bogotá Colombia dmendoza16@areandina.edu.co

**Keywords:** Quality of Life, Work-related Stress, Nursing, Covid-19, Pandemic, Calidad de Vida, Estrés Laboral, Enfermería, Covid-19, Pandemia, Qualidade de Vida, Estresse Relacionado ao Trabalho, Enfermagem, Covid-19, Pandemia

## Abstract

**Introduction:**

Work-related stress is the physical and emotional manifestation triggered by an imbalance in coping with perceived demands. One of the workers most affected by work-related stress is the nursing staff, a situation that was exacerbated during the COVID-19 pandemic and could affect their quality of life.

**Objective:**

To evaluate the connection between quality of life and work-related stress in nurses during the COVID-19 pandemic.

**Materials and Methods:**

Analytical, cross-sectional study, with a quantitative approach. Descriptive statistics and multiple linear regression analysis were used for the present study.

**Results:**

a negative correlation was obtained between quality of life and stress level (r = -0.438, p < 0.01), and being a professional nurse was related to higher stress levels. On the other hand, being 31 years or older and having more than 12 months of work experience were associated with a higher quality of life score, while being female, single, separated, or widowed and having higher acute stress scores indicated a significantly worse quality of life.

**Discussion:**

Acute stress negatively affects the quality of life of nurses during the COVID-19 pandemic and this differs significantly according to age, sex, marital status, and work experience.

**Conclusion:**

It is recommended that the health sector authorities design policies that guarantee strategies to improve the mental health of nursing personnel, as well as to guarantee fair and dignified treatment.

## Resumen

**Introducción:**

El estrés laboral es la manifestación física y emocional desencadenada por un desequilibrio en la capacidad de hacer frente a las exigencias percibidas1.Dentro de los trabajadores más afectados por el estrés laboral es el personal de enfermería2, situación que se exacerbó durante la pandemia por covid19 y pudo afectar la calidad de vida de estos.

**Objetivo:**

Evaluar la asociación entre calidad de vida y estrés laboral en enfermeros durante la pandemia por Covid19.

**Materiales y Métodos:**

Estudio analítico, transversal, con enfoque cuantitativo. Para el análisis se utilizaron estadísticas descriptivas y análisis de regresión lineal múltiple.

**Resultados:**

Se obtuvo una correlación negativa entre calidad de vida y nivel de estrés (r = -0,438, p < 0,01), así como ser enfermera profesional se relacionó con niveles más altos de estrés. Por otro lado, tener 31 años o más y más de 12 meses de experiencia laboral se asociaron con un mayor puntaje de calidad de vida, mientras que ser mujer, soltero, separado o viudo y puntuaciones mayores de estrés agudo indicaron una peor calidad de vida.

**Discusión:**

El estrés agudo afecta de forma negativa la calidad de vida de los enfermeros durante la pandemia por Covid19 y esta a su vez presenta diferencias significativas en función de la edad, sexo, estado civil y experiencia laboral.

**Conclusión:**

Se recomienda a las autoridades en el sector salud diseñar políticas que garanticen estrategias para mejorar la salud mental del personal de enfermería, así como garantizar trato justo y digno.

## Resumo

**Introdujo:**

O estresse no trabalho é a manifestado física e emocional desencadeada por um desequilíbrio na capacidade de lidar com as demandas percebidas. Entre os trabalhadores mais afetados pelo estresse no trabalho estáo os trabalhadores de enfermagem, situagáo que foi agravada durante a pandemia pela covid19 e pode afetar a qualidade da vida deles.

**Objetivo:**

Avaliar a associagáo entre qualidade de vida e estresse laboral em enfermeiros durante a pandemia de Covid19.

**Materiais e Métodos:**

estudo analítico, transversal, com abordagem quantitativa. Estatística descritiva e análise de regressáo linear múltipla foram utilizadas para a análise.

**Resultados:**

obteve-se correlagáo negativa entre qualidade de vida e nível de estresse (r = -0,438, p < 0,01), assim como ser profissional enfermeiro esteve relacionado a maiores níveis de estresse. Por outro lado, ter 31 anos ou mais e mais de 12 meses de experiencia profissional foram associados a uma maior pontuagáo de qualidade de vida, enquanto ser do sexo feminino, solteiro, separado ou viúvo e maiores pontuagóes de estresse agudo indicaram pior qualidade de vida.

**Discussao:**

O estresse agudo afeta negativamente a qualidade de vida dos enfermeiros durante a pandemia de Covid19 e este por sua vez apresenta diferengas significativas dependendo da idade, sexo, estado civil e experiencia profissional.

**Conclusao:**

Recomenda-se que as autoridades do setor da saúde desenhem políticas que garantam estratégias para melhorar a saúde mental do pessoal de enfermagem, bem como garantam um tratamento justo e digno.

## Introduction

Covid-19 is a disease caused by a new virus (SarsCov2) that originated in Wuhan, China, at the end of December 2019 and since then has spread rapidly to achieve viral spread throughout the world^1^.

In Colombia, 6,134,657 cases were reported until February 2022 and in Córdoba there was a decrease in the incidence of Covid-19 starting in July of this year, however, it has a cumulative total of 113,762 cases^2^. Of which the majority were reported in the municipality of Montería, capital of the department and where the main high level of complexity reference clinics are located. Now, the impact of the covid19 pandemic was initially presented as a health crisis that forced the restructuring of health models and care, which generated not only a crisis in the organizational management of health institutions but also in the staff. care that maintained direct contact in caring for patients in critical condition^3^.

In July 2021, in the city of Montería, a bed occupancy rate of 93.58% was reported, this being the third peak in the city, and in the fourth peak presented in February 2022, the occupancy was 7%. (5), at this point it is important to highlight that according to the Organization for Economic Cooperation and Development (OECD), for the year 2018 in Colombia there were 1.3 male and female nurses per 1,000 inhabitants^4^. situation that could have been exacerbated given the complexity of care for Covid-19 patients in critical condition, this being an influential factor in the work stress of nursing staff and is often related to the complexity of health care services^5^, In this case, hospital units where there are factors such as the suffering and pain of patients that can generate psychological stress and substantially affect the quality of life of the staff^6^.

Currently, it is possible to find various studies related to work stress in nursing staff, however, the impact that the Covid-19 pandemic could have on the quality of life of these staff and how this can generate a problem has not been studied long-term in public health. This is why the main objective of this research is to address the possible association between work stress perceived by nursing staff during the Covid-19 pandemic and quality of life, in such a way that public health strategies can be established and implemented to mitigate possible risk factors in nurses and also allow better work performance and quality care for patients.

## Materials and Methods

Analytical cross-sectional study with quantitative approach. The target population corresponds to the nursing staff who work in a fourth-level IPS (Institutional health service provider) in the city of Montería during the Covid-19 pandemic, this corresponds to 150 nurses and/or unit service nurses of intensive care unit (ICU), hospitalization, surgery, and emergencies. The study period corresponds to June 2021 until March 2022, data collection took place in February 2022 during the fourth peak of the pandemic in the study area.

Inclusion criteria: Work experience greater than 3 months, Work experience in the care area of emergency units, surgery, ICU, or hospitalization during the Covid-19 pandemic, exposure to direct contact with patients with a diagnosis of Covid-19 confirmed by RT-PCR or antigen. Exclusion criteria: History of disciplinary processes in the institution, History of psychological problems, death of family members or loved ones due to Covid-19 during the study period, and having been diagnosed with Covid-19 in the last 15 days. Non-probability convenience sampling was carried out.

Participants were informed about the voluntariness and anonymity of the test. The final sample was made up of 125 people, corresponding to 96% of the population, of which 84.8% were women. 52% of the participants are between 18 and 30 years old, married or free union marital status predominated in 52%.

A survey was applied where potential participants were recruited exclusively online by sending a link to a Google form that contained the explanation of the study, informed consent and a survey divided into 3 parts: the first consisted of a survey on sociodemographic questions, the second part consisted of a questionnaire on the level of work stressors based on the Nursing Stress Scale (NSS) validated in the Spanish language^7^ and in Colombia with a Cronbach's alpha 0.89^7^ and the third part of the survey consisted of the quality of life questionnaire (WHOQOL-BREF) which is also validated in Colombia, Cronbach's alpha 0.8^8^.

The instruments used were:

- Sociodemographic Questionnaire: contained variables such as age, sex, marital status, educational level, work experience, length of service, type of contract, position, number of dependents, number of monthly hours worked in the last month, service in which they work, as well as questions aimed at verifying compliance with the inclusion and exclusion criteria.
- NSS: consists of 34 questions grouped into 7 areas of work stress (F1=7 items, death and suffering, F2=5 items conflict with doctors, F3=3 items inadequate training, F4=3 items lack of support, F5=5 items conflict with other nurses F6=6 items workload, F7= 5 items uncertainty about treatment) which in turn are classified into 3 spheres, physical (workload), psychological (death and suffering, inadequate training, lack of support and uncertainty about treatments) and social (conflict with doctors and conflict with other nurses).
- Responses on the NSS are based on a Likert-type scale with 4 response options (never=0, sometimes=1, frequently=2, and very frequently=3) and results in an overall score that ranges from 0 to 102, the higher the score is associated with a higher level of stressors^9^.
- The WHOQOL-BREF questionnaire: consists of 26 Likert-type questions with a response option between 1 and 5 with a total score ranging between 26 and 130 and includes 2 questions on perception of quality of life and health, in turn it is classified into 4 domains. (physical, psychological, social relationships and environment) for each domain the scores can be between 4 and 20 or they can be transformed to a scale of 0-100 points to equate the scores to the WHOQOL-100 (original instrument)^10^ and higher scores reflect a better quality of life^11^.

A descriptive analysis of statistics was carried out for all variables using the IBM SPSS version 27 program for Windows, as well as sociodemographic data analysis with measures of central tendency such as the mean and standard deviation, absolute and relative frequencies to summarize categorical variables and define whether the differences between one variable and another were really significant. Likewise, the Kolmogorov Smirnov test was used to evaluate normality, observing that the data have a normal distribution (p>0.05). Differences in means analysis was carried out using the t student test for dichotomous variables, with those with significant p value <0.05, as well as Levene's test for difference of variances, for the polychotomous variables an ANOVA test was performed and the Post hoc values were reported as significant p<0.05 (in cases where there was no post hoc, only the p values were reported. The correlation between the level of stress of the nursing staff and the quality of life was estimated through Pearson’s bivariate correlation analysis and bivariate linear regression analysis was performed in order to identify possible associations between the sociodemographic variables and the stress levels as well as with the quality of life individually, subsequently the variables with individual significance p=<0.05 were entered into the multiple regression model taking into account the quantitative dependent variable quality of life. The database is publicly accessible at Mendeley Data^12^.

The researchers declare that they have a conflict of interest since they currently work in the institutions where the study will be carried out. However, this did not affect the transparency in the collection and processing of the research. Control was carried out by blinding the researcher who works in the institution, which granted endorsement by the ethics committee. Likewise, the recommendations for research related to the health of human beings prepared by the Council of International Organizations of Medical Sciences (CIOMS) were taken into account^13^. Therefore, the work was submitted to the ethics committee after intervention. The Declaration of Helsinki was also taken into account^14^, through which the ethical principles for medical research on human beings and the Belmont report are established^15^, where the principles and ethical guides for the protection of human subjects in research are established.

## Results

According to the descriptive analyses, when evaluating the sociodemographic characteristics that can be observed in Table 1, it was obtained that 52.22% (65) of the participants are between 18 and 30 years old, the sample was made up by women representing 84.78% (106), with a predominance of married or free union marital status, depending on their position in 81.64% (102) as nursing assistants and 18.46% (23) correspond to professional nurses.

Regarding work experience, 80.88% (101) of the participants had more than 12 months. See Table 1.

**Table 1:** Sociodemographic and labor characteristics of the population under study

Variable	Categories	n	%	Stress level			Quality of life		
				- *IX* ± SD	t/F	p value	- *IX* ± SD	t/F	p value
Age (Years)	18 to 30 years	65	52.22	45.11 ± 14.30	0.232	0.817	61.26 ± 12.34	-1,869	0,064
≥ 31 years	60	47.78	44.52 ± 14.15	65.45 ± 12.74
Sex	Man	19	15.22	46.63 ± 17.58			69.29 ± 13.04		
	Women	106	84.78	44.5 ± 13.55	0.602	0.548		2,202	0,024
Civil status	Married-free union	65	52.22	42.46 ± 14.18	-1.961	0.052	66.78 ± 11.8	3,355	0,001^*^
	Single-divorced-widowed	60	47.78	47.38 ± 13.83			59.47 ± 12.55		
Number of dependents (n)	1 or less	41	32.88	43.46 ± 12.03			64.56 ± 13.19	0.365	0.695
	2 to 3	68	54.44	44.85 ± 15.4			62.87 ± 12.4		
	4 or more	16	126.8	48.19 ± 14.10			61.70 ± 12.89		
Position	Nursing assistant	102	81.64	43.43 ± 13.83	0.636	0.531	64.09 ± 12.26	1.520	0.131
	Professional nurse	23	18.46	51 ± 14.35			59.67 ± 14		
Work experience	≤12 months	24	19.22	43.8 ± 15.52	-0.668	0.506	58.00 ± 10.43	-2.309	0.023^*^
	> 12 months	101	80.88	45.24 ± 15.52			64.53 ± 12.86		
Type of contract	Fixed term contract	53	42.44	43.94 ± 14.49	-0.594	0.553	62.45 ± 12.01	-0.622	0.535
	Indefinite-term contract	72	57.66	45.47 ± 14.01			63.88 ± 13.17		
Number of monthly hours worked last month	≤ 192 hours per month	25	20.22	42.68 ± 14.75	-0.845	0.400		-1.866	0.064
	> 192 hours per month	100	80.88	45.36 ± 14.05			64.32 ± 13,0		
Service in which they currently work	Emergencies	4	3.25	53.5 ± 7.9	0.590	0.623	52.93 ± 6.63	2.460	0.066
	Hospitalization	35	28.23	43.69 ± 16.95			64.37 ± 12.17		
	ICU	81	64.81	44.79 ± 13.2			64 ± 12.43		
	Surgery	5	4.05	46.4 ± 13.01			52.05 ± 17.26		

(X) = mean, SD = standard deviation ^*^p<0.05: significant difference. ^+^p test value from Gabriel, ^++^p test value from Games Howel

In the univariate analysis, in relation to the level of acute stress, significant differences in position were observed. Acute stress was significantly higher in professional nurses (t=-2.355, p value=0.020) with a mean of 51 and SD 14.35 than in nursing assistants whose mean was 43.43 and SD 13.83. In the other variables, no significant differences were observed in relation to the level of stress (p value>0.05).

Regarding quality of life, significant differences were observed in sex (t=2.202, p value=0.024), marital status (t=3.355, p value=0.001) and work experience (t= -2.309, p value p=0.023). Regarding sex, men had a better quality of life (X=69.29 SD=13.04) than women (X=62.19 SD=12.34), in the married or free union marital status, the quality of life was higher (X=66.78 SD=11.8) than, in single, divorced or widowed people (X=59.47 Sd=12.55) and those with work experience greater than 12 months, higher quality of life scores were also observed (X=64.53 SD=12.86) (Table 1).

### Correlation between quality of life measured through the Whoqol-Bref and level of acute stress measured through NSS

In Table 2 it can be seen that the response to acute stress of the study participants was negatively correlated with quality of life (r=-0.438, p < 0.01). Furthermore, the 3 spheres of the NSS were significantly and negatively correlated with almost all dimensions of quality of life except for the correlation between physical environment and social relationships where the correlation was not significant (p>0.05), this determines the strong negative association between quality of life and the stress levels, with a higher level of stress decreasing the quality of life of the nurses in the study.

**Table 2:** Pearson correlation analysis between NSS and WHOQOL-Bref

Variables	Total Score Whoqol-Bref	Physical	Psychological	Social relationships	Environment
Total Score NSS	-.0438^**^	-.0480^**^	-0.370^**^	-0.255^**^	-0.421^**^
Physical environment	-0.290^**^	-0.347^**^	-0.216^*^	-0.153	-0.306^**^
Psychological environment	-0.373^**^	-0.402^**^	-0.318^**^	-0.187^*^	-0.410^**^
Social environment	-0.435^**^	-0.469^**^	-0.384^**^	-0.314^**^	-0.318^**^

Whoqol-Bref, quality of life. NSS (Nursing Stress Scale). ^*^p<0.05 (2-tailed) ^**^p<0.01 (2-tailed) based on Pearson’s correlation coefficient.

### Factors influencing the quality of life and acute stress of the nurses in the study

For the dependent variable quality of life, all independent variables of the research were included, mainly performing a bivariate analysis, and those with significance values (p<0.05) were included in the final model developed using the backward method. The variable level of stress, age, sex, marital status, number of dependents, position within the institution, work experience, number of monthly hours worked and service in which they work were mainly taken into account, however, when running the final multiple linear regression model, only the variables stress level, age, sex, marital status and work experience were significant (p<0.05), observing that for each point that the stress level increases, the quality of life decreases by 0. .3 points (B= -0.360, p=<0.001), being 31 years old or older increases quality of life by 4 points compared to people aged between 18 to 30 years (B=4.081 p=0.033), being a woman reduces quality of life by 6.5 points compared to men (B= -6.581, p=0.013), being single- divorced-widowed reduces quality of life by 5.7 points compared to married- free union marital status (B=-5.744, p=0.003), having more than 12 months of work experience increases quality of life by 5.9 compared to people who have work experience < or equal to 12 months ( B=5.931, p=0.015). These 5 variables collectively and statistically significantly predicted quality of life by 39% (R2 =0.390, p<0.05). It is important to highlight that the level of stress accounted for most of the variance at 19.2% (R2 =0.192, adjusted R2=0.185). However, with the variables included in the research, only 39% of the quality of life variable can be explained, therefore it is necessary to continue developing research related to the topic where it is possible to link a greater number of variables such as job satisfaction, and comorbidities that can significantly influence quality of life.

Based on the above results, the following equation resulting from the multiple linear regression model can be proposed, that collectively explains 39% of the quality of life variable, so it could then be stated:

Quality of life= 82.5+4.081 (age > 31 years)-6.581 (female sex)-5.744 (marital status: single-divorced or widowed) +5.931 (work experience 12 months)-0.360 (stress level). (Value of the variables equation =1)

**Table 3:** Multiple linear regression analysis of the factors influencing stress at work

Variable	Categories	Unstandardized coefficients (B)	Std. Error (SE)	Standardized coefficient (β)	t	p value	Confidence interval for B	
							Lower limit	Upper limit
Age (Years)	18 to 30 years	1						
> 31 years	4.081	1.893	0.162	2.156	0.033^*^	0.333	7.829
Sex	Man	1						
	Woman	-6.581	2.618	-0.187	-2.514	0.013^*^	-11.766	-1.396
Civil status	Married-free union	1						
	Single-divorce-widowed	-5.744	1.899	-0.228	-3.025	0.003^*^	-9.504	-1.983
Work experience	≤12 months	1						
	> 12 months	5.931	2.402	0.185	2.469	0.015^*^	1.173	10.688
Stress level (SNS)		-0.360	-0.360	0.066	-0.403	-5.441	<0.001^**^	-0.490

NSNSS, Nursing Stress Scale, explanation of the model based on the level of stress (R2 =0.192, adjusted R2=0.185), explanation of the general model (R2 =0.390, adjusted R2=0.354). ^*^p<0.05 ^**^p<0.01

After performing the multiple linear regression analysis to identify the factors that influence quality of life including the level of stress, another model was carried out for the acute stress level variable in order to identify the sociodemographic variables that may be directly related to the level of stress and could have generated a bias in the model, taking as references those with a value of p < 0.05 and in Table 3 shows the result obtained, in which the only variable that was significantly associated with the level of stress was the position (Professional nurse B=7.569 p=0.020), meaning that being a professional nurse increases the level of stress by 7.5 points on the NSS scale and this variable represents 3.5% of the variance of the NSS (R2 =0.043, adjusted R2=0.035). Given that the variables included in the research only explain a minimal part of the acute stress variable, it is possible that other variables with a higher explanatory level, such as physical or psychological comorbidities, were not considered in the research, therefore further research is required.

**Table 4:** Multiple linear regression analysis of the factors that influence the response to acute stress

Variable	Categories	Unstandardized coefficients (B)	Std. Error (SE)	Standardized coefficient (β)	t	p value	Confidence interval for B	
							Lower limit	Upper limit
Post	Nursing assistant	Reference						
Professional nurse	7.569	3.214	0.208	2.355	0.020^*^	1.207	13.930

Explanation of the level of stress from the position (R2 =0,043, adjusted R2=0,035) ^*^p<0.05 ^**^p<0.01

**Figure 1:**
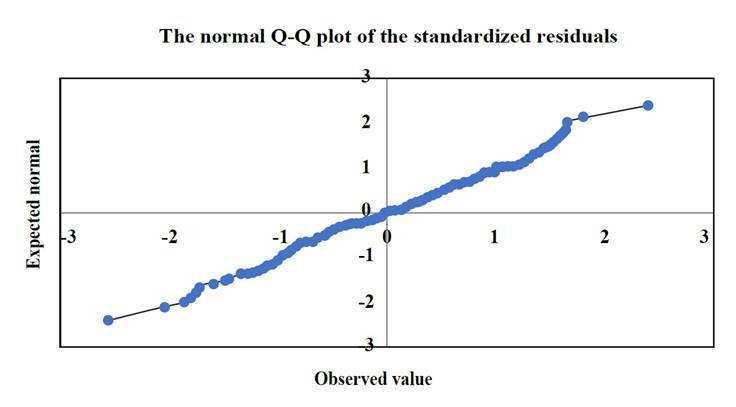
Normality of data SPSS version 26, Kolmogorov Smirnov normality test p value =0.2

A report is made on the assumption of normality of the data analyzed (p=0.2). In each of the models, the assumptions of normality, homoscedasticity, independence with a Durbin-Watson of 1.98 were evaluated and found to be satisfactory, and no multicollinearity problems were observed (tolerance>0.9). See Figure 1.

## Discussion

The objective of our study was to determine the association between work-related stress and quality of life in nursing staff during the Covid-19 pandemic in a highly complex IPS (health services provider institution), finding that the response to acute stress ofthe participants was negatively correlated with quality of life (r = -0.438, p < 0.01) and compared to sociodemographic characteristics in the univariate analysis the variables sex, marital status and work experience showed significant differences (P < 0.05) in the levels of quality of life, likewise in the multivariate analysis the relationship between the level of stress and quality of life was demonstrated, being that for each point that the level of stress increases, the quality of life decreases by 0.3 points (B = -0.360, P=<0.001).

Considering the characterization of the study population, it was observed that the nursing group is represented mostly by women. In Colombia, according to the national policy of human talent in health, the profound feminization of health personnel stands out: three quarters are women. and, except in medicine, in all health professions and occupations a higher proportion of women is observed, being particularly high at the auxiliary. In addition, with respect to age, 47.9% of the estimated available human talent corresponds to people under 35 years of age, while 14.1% corresponds to people over 49 years of age^16^. These data are similar to those found in a study carried out in Peru in a III level hospital aimed to evaluate the levels of anxiety, depression and stress in emergency nurses during Covid-19, finding that 39.1% of the nurses presented some level of anxiety, 24.6% some level of depression and 8.8% some level of stress, likewise the majority were women (61.9%), 42.1% had a permanent contract and 38.9% had more than 1 year from experience^17^.

Regarding the position, it can be seen in table N.1 that 81.6% ofthe participants are nursing assistants and 18.4% are nursing professionals. When reviewing the national policy on human talent in Nursing^18^ of the total number of workers in the health sector, 66.095 corresponded to nursing professionals and 273.359 to nursing assistants, which proves that the auxiliary nursing staff is greater throughout the Colombian territory.

The demographic characterization of our participants is very similar to what was found in a study called work stress in nursing and associated factors^19^, carried out in the city of Cartagena which showed that the average age was 33.2 years (SD= 8.4), of these 147 (94.2%) were women, in addition 93 (59.6%) of the respondents had a stable partner and a median number of dependents of 2.

In the univariate analysis of the present study, it was observed that there are significant differences between nursing assistants and professional nurses in relation to the level of stress, being higher in the latter group (t=-2.355, p value=0.020), this is related to other research consulted^20^^,^^21^. Regarding quality of life, significant differences were observed in sex (t=2.202, p value=0.024), marital status (t=3.355, p value=0.001) and work experience (t= -2.309, p value p=0.023) this is related to what was found in the systematic review on quality of life of health personnel during the Covid-19 pandemic^22^, in which 5 studies indicated a relationship between age, female sex and work experience on the prevalence of anxiety and stress levels of health personnel during the covid-19 pandemic.

On the other hand, in our research a significant negative correlation was observed between work- related stress and quality of life (r = -0.438, p < 0.01), which is associated with what was found in the study called “Burnout syndrome, stress work and quality of life in nursing workers” in which they conclude that nursing professionals had high levels of occupational stress and this is negatively related to the perception of quality of life (r= -0.035, p value <0.01)^23)^and in other investigations, similarities were also found in the negative correlation between quality of life and work-related stress in nursing (r= -3.045 p value= <0.05)^24^.

These results also agree with those found in the study carried out at the Catholic University of Santiago de Guayaquil called "Work-related stress and quality of work life of the nursing professional at the Hospital de EspecialidadesTeodoro Maldonado Carbo in which it was observed that the greater the stress work, the lower the quality of life (r= -0.563, p value <0.01)^25^observing a greater negative correlation in relation to the results obtained in our research.

In the multiple linear regression analysis on the dependent variable quality of life, it was observed that being 31 years old or older (B=-5.744, P=0.003), being married or in a free union (B=-5.744, P=0.003) and having more than 12 months of work experience (B=5.931, P=0.015) were significantly related to better quality of life. The above is compared with the study called “Relationship Between Acute Stress Responses and Quality of Life in Chinese Health Care Workers During the COVID-19 Outbreak” in which quality of life was significantly related to the level of stress (β = − 0.545, p < 0.001) and ages over 35 years (β = 0.143, p < 0.001)11 and in terms of marital status, they found that widowed or divorced people reported responses of worse quality of life, which is related to Pérez et al. ^26^ who stated that divorce or the loss of a spouse is usually accompanied by numerous negative consequences and psychological distress. Likewise, the variable that was significantly associated with the level of stress in our research was the position (professional nurse B=7.569 p=0.020), coinciding with multiple investigations related to exercise and compassion fatigue of professional nurses and high levels of stress^6^^,^^27^^,^^28^^,^^29^.

Finally, in the study it was possible to identify that the position is a factor that is directly related to work stress and to a greater extent when it comes to nursing professionals and as well as variables that are 39% related to quality of life of the nursing staff and they are > 31 years old and this can be explained because at older age there may be a greater ability to cope with situations that generate uncertainty or stress^30^, being a woman, marital status single-divorced or widowed, work experience greater than 12 months, possibly because having more experience means greater capacity to resolve risks.^31^ especially in the ICU staff who constitute 64.8% of the staff surveyed, and the level of stress, the latter of which individually explains 19% of the dependent variable quality of life.

In general terms, our research can contribute to identifying the possible factors that affect the quality of life of workers and therefore their work performance and to design strategies by managers of health institutions or health secretaries to face these situations both in the Covid-19 pandemic as well as in future pandemics that may occur. However, it is important to highlight that there are some limitations of the research such as the self-administered survey to the participants due to the high exposure to the virus when being in contact with distances less than 2 meters, the adaptation of the survey questions taking into account the Covid-19 pandemic, which could lead to memory biases, furthermore, since it was a cross-sectional study, the levels of stress or quality of life were not re-evaluated to investigate differences, nor was a baseline of the stressors that could be presented prior to the Covid-19 pandemic. Similarly, a more representative sample is required to generalize our research. It is important to note that the research group proposed the development of the research in a multicenter manner but approval was not obtained from the other institutions, so it is also important that in the future various institutions are taken into account and evaluate the differences or similarities that may be found. On the other hand, a bias in measurement is declared in the research because the data were obtained in the fourth peak of the pandemic approximately 11 months after the first case presented, so the results could vary if retrospective values had been available.

## Conclusions

After analyzing the results, it is concluded that acute work-related stress has a negative correlation with the quality of life variable, finding that stress negatively affects most dimensions of quality of life, except for the physical environment and social relations where the differences found were not significant. The stress level variables were significantly related to the position they hold in the institution, especially when it is a nursing professional. Moreover, being over 31 years old, being a man, being married or in a free union and having more than 12 months of work experience are factors that increase the quality of life. On the other hand, for every point that the stress level increases, the quality of life decreases by 0.3.

The results of this study can be useful to improve aspects such as the work environment, promoting the creation of mental health activities and work well-being. In addition, decision makers should design policies so that health workers can obtain equitable salary compensation, improve the contracting method, and receive fair and dignified treatment.
